# Malignant ovarian dysgerminoma in a 16-year-old leopard gecko (*Eublepharis macularius*)

**DOI:** 10.17221/107/2022-VETMED

**Published:** 2023-03-22

**Authors:** Henrieta Zborilova, Janosch Dietz, Kim Oliver Heckers, Radka Dvorakova, Zdenek Knotek, Eva Cermakova

**Affiliations:** ^1^Avian and Exotic Animal Clinic, Faculty of Veterinary Medicine, University of Veterinary Sciences Brno, Brno, Czech Republic; ^2^Department of Pathology, LABOKLIN Laboratory for Clinical Diagnostics GmbH & Co. KG, Bad Kissingen, Germany; ^3^Department of Imaging Methods, Dog and Cat Clinic, Faculty of Veterinary Medicine, University of Veterinary Sciences Brno, Brno, Czech Republic

**Keywords:** histopathology, lizards, malignant neoplasia, surgery, reptiles

## Abstract

The 16-year-old female leopard gecko (*Eublepharis macularius*) was presented with distended coelom and cachexia. Examination of the faecal sample ruled out the presence of protozoan parasites. A radiographic examination confirmed the presence of radiopaque foreign material in the intestine. The conservative treatment with tramadol, butylscopolamine, famotidine, vitamin B complex, and supportive fluid therapy with Hartmann solution and Duphalyte, was performed for 14 days. Ultrasonographic examination revealed the presence of a large mass adherent to the liver (with hypoechoic regions), a thin-walled cystic structure close to the liver, and coelomic effusion. Surgical exploration revealed a large mass on the right ovary. The unilateral (right) ovariectomy was performed. Histologic examination of the mass revealed dysgerminoma with an invasion of the ovarian bursa and blood vessels. Nine months after the surgery the patient was active and doing well. In reptiles, dysgerminoma is an uncommon type of neoplasia. To the best of our knowledge, this is the first case of dysgerminoma tumour diagnosed intravitally and treated successfully in lizards.

Based on their popularity as pets and the increased frequency of visits to veterinarians, lizards are over-represented in recent clinical case reports (Christman et al. 2017). They are presented to veterinary clinics with a broad spectrum of diseases, including tumours.

The standard protocol of reptile patient clinical examination consists of physical examination, radiographic and/or ultrasonographic examination, faecal samples examination, and analyses of haematology and plasma chemistry profiles (Schumacher and Toal 2001; Morici et al. 2018; Cermakova et al. 2019; Divers 2019; Knotkova et al. 2019; Mayer and Moore 2019).

Surgically excised tissues should always be submitted for histopathologic analysis.

## Case description

A 16-year-old female leopard gecko (*Eublepharis macularius*), with a body weight of 52 g and body condition score of 1/5, was presented at a clinic with distended coelom and a history of weight loss within the last 6 weeks. According to the owner, the patient had no problems with appetite, urination, or defaecation, and the animal was otherwise considered healthy. The female was kept individually, under 12 h light/12 h dark photoperiod, in a terrarium with sand as the substrate. The temperature within the enclosure was 25 °C to 28 °C. The diet consisted of crickets and occasionally mealworms.

**Figure 1:**
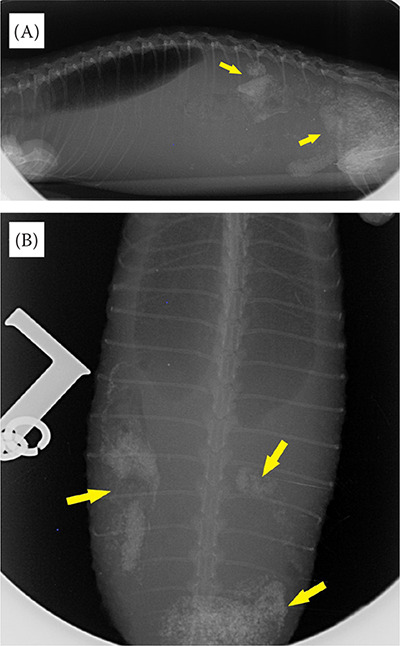
Radiography of female leopard gecko Standard projections DV (A) and LL (B) revealed radiopaque content in the intestines of the patient (yellow arrows) DV = dorsoventral; LL = laterolateral

**Figure 2:**
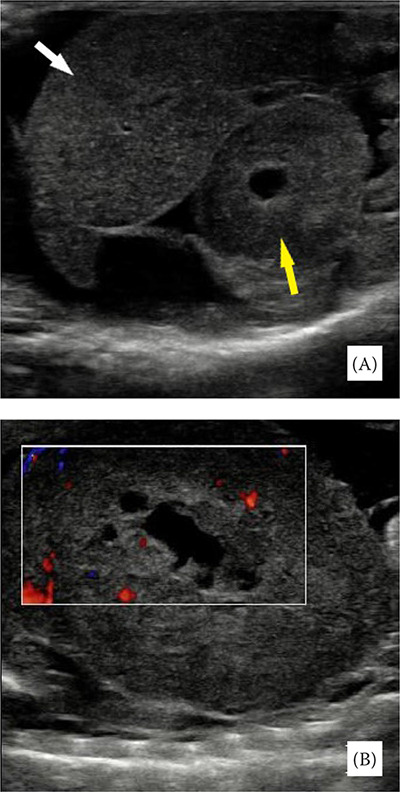
Ultrasonography of female leopard gecko (A) The cranial part of the mass (yellow arrow) was situated near to the liver (white arrow). Echogenicity and echotexture of the liver tissue and the mass were similar. (B) Blood supply of the mass (red colour)

Physical examination revealed large distention of the coelom. The oral mucosa was lighter in colour. The patient was calm but responsive. Radiographic examination due to the size of the patient was performed in standard dorsoventral (DV) and laterolateral (LL) projections by intraoral dental X-ray system Gendex expert DC (Gendex Dental Systems; Des Plaines, IL, USA; indirect imaging by CR 7 Vet; Dürr Medical, Bietigheim-Bissingen, Germany). Radiographs revealed intestines filled with radiopaque content (Figure 1A,B). Haematology and plasma chemistry examinations were not performed because of the poor body condition score of the patient. The patient was hospitalized. As a conservative treatment, the patient received daily tramadol (10 mg/kg, Tramal; Stada, Bad Vilbel, Germany), butylscopolamine (5 mg/kg, Buscopan; Ipsen, Boulogne-Billancourt, France), famotidine (1 mg/kg, Quamatel; Gedeon Richter Plc. Budapest, Hungary), vitamin B complex (10 mg/kg, Milgamma N; Worwag Pharma GmhH and Co. KG, Böblingen, Germany) intramuscularly. A mixture of Hartmann solution and Duphalyte (20 ml/kg, ratio 4 : 1, Hartmann Solution; B. Braun Deutschland GmbH & Co. KG, Melsugen, Germany; Duphalyte; Zoetis Manufacturing & Research, Grona, Spain) was administered subcutaneously. Since the second day of the treatment patient defeacated spontaneously with a small amount of sand in the faeces. Examination of the faecal sample ruled out the presence of any gastrointestinal parasites. The patient was regularly fed with a commercial diet (Emeraid Intensive Care Carnivore; EmerAid LLC, Cornell, IL, USA), enriched with immunoglucans (Imunoglukan P4H; Pleuran, Bratislava, Slovakia), carnitine (Vigosine; CEVA Animal Health, Bratislava, Slovak Republic) and a small amount of paraffin oil (Fagron a.s., Olomouc, Czech Republic) or cannabis oil (Cannasan; Research Institute Cannasan, Slušovice, Czech Republic). Despite the treatment and spontaneous defaecation, the distension of coelom persisted. Ultrasonographic examination (Ultrasound system RS85; Samsung Medison Co. Ltd., Seoul, Republic of Korea; linear probe 3–16 MHz), performed after two weeks of conservative treatment, revealed a large mass with hypoechoic regions adherent to the liver, thin-walled cystic structure close to the liver and presence of coelomic effusion (Figure 2A,B), confirming the need for surgical intervention.

**Figure 3:**
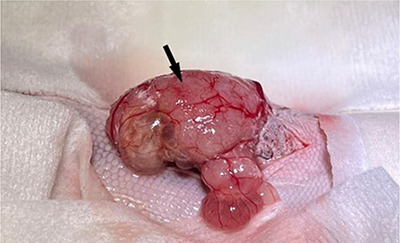
Female leopard gecko – right ovary with the tumour mass (black arrow)

**Figure 4:**
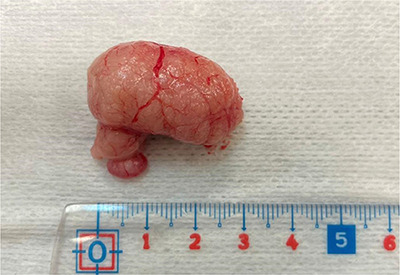
Female leopard gecko – excised tumour mass

**Figure 5:**
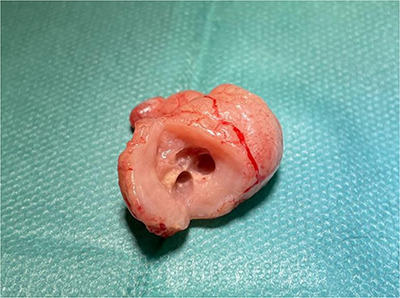
Cross section through the mass

Premedication before surgery consisted of me-loxicam (1 mg/kg, Melovem; Dopharma Research B.V., Raamsdonksveer, The Netherlands) and tramadol (10 mg/kg, Tramal; Stada, Bad Vilbel, Germany) intramuscularly. Forty-five minutes later, alfaxalone (10 mg/kg, Alfaxan; Jurox Pty Limited, Rutherford, NSW, Australia) was administered intramuscularly. An endotracheal tube (Vasofix Safety G20; B. Braun Deutschland GmbH & Co. KG, Melsugen, Germany) was placed and anaesthesia was maintained with isoflurane (2–3% isoflurane, Aerrane; Baxter, Lessines, Belgium) in oxygen (0.5 l/minute). A standard celiotomy was performed on the patient in dorsal recumbency. A relatively well-defined mass was attached to the right ovary, partially adhered to the hepatic lobe (Figure 3). The mass (6.23 g) and the right ovarium were completely ligated with resorbable polyphilament suture material (PGA 4/0; Resorba Medical GmBH, Nürnberg, Germany) and removed (Figures 4 and 5). Small white dots (< 1 mm in diameter) were observed on the surface of the pleuroperitoneum and serosal covering of internal organs. The pleuroperitoneum with muscular layer and skin were closed separately with resorbable monofilament material (PDO 4/0; Resorba Medical GmBH, Nürnberg, Germany) according to the standard protocol. Post-operatively and within the next seven days, marbofloxacin (10 mg/kg, Quiflox 2%; Krka d.d., Novo Mesto, Slovak Republic) was daily administered intramuscularly. Meloxicam was administered daily for a total of 5 days and tramadol for a total of 2 days. Since the 9^th^ day, the patient started to eat spontaneously. Check-ups and suture removal were performed six weeks after surgery. Nine months after the surgery, the patient is active and doing well.

**Figure 6:**
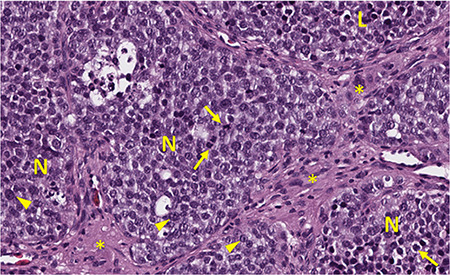
Histopathology

The excised mass was submitted for histopathological examination. A mass originating from germ cells was found. The cells were large, round to oval, and had an abundant amount of clear amphiphilic cytoplasm. Anisocytosis, anisokaryosis the mitotic index was moderate. Some blood vessels showed invasion of the tumour cells. The margins of the tissue were not clean, because the tumour cells invaded the organ capsule. Histological examination revealed dysgerminoma with the invasion of the ovarian bursa and blood vessels (Figure 6).

Sheets of large neoplastic round cells (N) in a scant amount of fibrovascular stromal tissue (*). The cells were round to oval with a centrally located round nucleus with granular chromatin and 1–2 prominent nucleoli (arrowhead). Anisocytosis and anisokaryosis were mild to moderate. Multiple mitotic figures (arrow) were seen. Low numbers of lymphocytes (L) were seen multifocally

## DISCUSSION

In old reptiles, neoplasia should always be considered a differential diagnosis (Hedley 2016). Clinical signs vary depending on tumour type and its location, therefore a broad complex of diseases including neoplasia have to be considered in patients suffering from anorexia and lethargy. In reptiles diagnosed with neoplasia, the most common symptoms include lethargy, anorexia, dyspnoea, presence of cutaneous masses, coelomic distension, constipation, or paresis/paralysis (Hernandez-Divers and Garner 2003; Cruz Cardona et al. 2011; Hannon et al. 2011; Heckers et al. 2012). Based on a necropsy review, captive reptiles have an incidence of neoplasia comparable with that of mammals and birds (Effron et al. 1997). In lizards, neoplasms appear to arise very often from the haematopoietic system, skin, and liver (Hernandez-Divers and Garner 2003). Tumours of the reproductive system are common in reptiles, especially in lizards and snakes (Garner et al. 2004). The most common reproductive tumour in lizards and snakes is ovarian adenocarcinoma (Gibbons and Schiller 2000; Stacy et al. 2004; Sykes and Trupkiewicz 2006). Primary ovarian neoplasms are classified into the three groups based on the cells of origin: epithelial tumours (adenocarcinoma and adenoma), germ-cell tumours (teratoma and dysgerminoma), and sex cord tumours (granulosa theca cell tumour, thecoma, granulosa-theca cell, and lutheoma) (MacLachlan and Kennedy 2002). Ovarian teratomas were reported in lizards, especially in green iguanas (Anderson et al. 1996; Bel et al. 2016). While ovarian carcinomas, fibromas, and hemangiomas have been reported frequently in reptiles (Frye 1994; Garner et al. 2004; Sykes and Trupkiewicz 2006), dysgerminoma has been reported in only two cases in chelonians (Machotka et al. 1992; Frye et al. 1998). Dysgerminoma is a germ cell tumour that arises from undifferentiated pluripotent germ cells of the gonad epithelium and is the counterpart of the testicular seminoma. In domestic species, this type of tumour is relatively rare compared to the other types of reproductive tract tumours, except for the testicular seminoma in dogs (Grieco et al. 2008). These tumours are usually malignant and locally invasive, they can grow to large sizes. Clinically, they can develop signs of body wall distension followed by coelomic discomfort usually accompanied by anorexia and in some cases in some cases by dyspnoea. In mammals, hormonal disbalance such as persistent oestrus was described, because the tumour can produce oestrogen (Klein 2007). The mass can invade via the surrounding bursa and the blood or lymph vessels. It can develop coelomic implantation metastases or metastases to the other parenchymatous organs. Diagnosis of dysgerminoma is based on clinical examination with the use of diagnostic imaging methods (radiography, ultrasonography) followed by cytological or histopathological diagnostic techniques. Treatment includes surgical excision of the mass (followed by radiation and chemotherapy in humans). The prognosis of dysgerminoma depends on the development of metastases (Novotny et al. 2011). Dysgerminomas have been reported in birds, reptiles, amphibians, Chondrichthyes, and fish – eastern rosella (*Platycercus eximius*; Strunk et al. 2011), snapping turtle (*Chelydra serpentina*; Machotka et al. 1992), red-eared terrapin (*Trachemys scripta elegans*; Frye et al. 1998), mountain chicken frogs (*Leptodactylus fallax*; Fitzgerald et al. 2007), orange-spot freshwater stingray (*Potamotrygon motoro*; Jafarey et al. 2015) and largemouth bass (*Micropterus salmoides*; Masahito et al. 1984).

To the authors’ knowledge, this is the first report of ovarian dysgerminoma in lizards. This finding has a significant impact on the differential diagnosis of neoplasms of the reproductive system in reptiles.
